# Prevalence of and risk factors for diabetic retinopathy in residents with different types of abnormal glucose metabolism with or without hypertension: A suburban community-based cross-sectional study

**DOI:** 10.3389/fendo.2022.966619

**Published:** 2022-08-08

**Authors:** Yuhang Ma, Hao Wang, Junyi Jiang, Changjing Han, Chunhua Lu, Siliang Zeng, Yufan Wang, Zhi Zheng, Yongde Peng, Xiaoying Ding

**Affiliations:** ^1^ Department of Endocrinology and Metabolism, Shanghai General Hospital, Shanghai Jiao Tong University School of Medicine, Shanghai, China; ^2^ Key Laboratory of Ophthalmology, Department of Ophthalmology, Hebei Eye Hospital, Xingtai, China; ^3^ Department of Clinical Pharmacology, Xiangya Hospital, Central South University, Changsha, China; ^4^ Department of Ophthalmology, The Second Affiliated Hospital of Xi’an Jiao Tong University, Xi’an, China; ^5^ Preventive Medicine Division, Community Health Service Center of Sijing, Shanghai, China; ^6^ Department of Rehabilitation Therapy, School of Health, Shanghai Normal University Tianhua College, Shanghai, China; ^7^ Shanghai Key Laboratory of Ocular Fundus Diseases, Department of Ophthalmology, Shanghai General Hospital, Shanghai Engineering Center for Visual Science and Photomedicine and Shanghai Engineering Center for Precise Diagnosis and Treatment of Eye Diseases, National Clinical Research Center for Eye Diseases, Shanghai, China

**Keywords:** prevalence, risk factors, diabetes, diabetic retinopathy, hypertension

## Abstract

**Aims:**

The present study examined the prevalence and risk factors for diabetic retinopathy (DR) in residents with abnormal glucose metabolism in a community.

**Methods:**

6029 subjects were included and underwent standardized interviews and comprehensive examinations. Residents with diabetes were divided into nondiabetic retinopathy (NDR) and DR groups and non-hypertension and hypertension groups. Unconditional multivariate logistic regression models were used to analyze the risk factors for DR in different groups.

**Results:**

The prevalence of DR in diabetes was 9.9%, and the prevalence of retinopathy, which also has the typical signs of DRs, such as retinal microaneurysms, in prediabetes and normal glucose tolerance was 5.2% and 5.3%, respectively. An elevated waist-to-hip ratio (WHR) (female≥0.85, male≥0.9)[OR 1.683, 95% CI (1.016, 2.790)], systolic blood pressure (SBP)≥140 mmHg [OR 1.875, 95% CI (1.158, 3.034)], elevated HbA1c [OR 1.410, 95% CI (1.220, 1.629)], HbA1c ≥6.5% [OR 2.149, 95% CI (1.320, 3.498)], antidiabetic drug use [OR 3.798, 95% CI (2.209, 6.529)], elevated fasting blood glucose [OR 1.176, 95% CI (1.072, 1.289)], elevated postprandial blood glucose [OR 1.090, 95% CI (1.033, 1.150)] and nonspecific ST-T segment changes on electrocardiography [OR 2.555, 95% CI (1.556, 4.196)] were risk factors for DR. Duration of diabetes [OR 1.206, 95% CI (1.028, 1.415)], elevated WHR [OR 3.796, 95% CI (1.144, 12.603)], elevated waist circumference [OR 6.874, 95% CI (1.403, 33.665)], elevated HbA1c [OR 1.435, 95% CI (1.046, 1.970)], HbA1c ≥6.5% [OR 6.850, 95% CI (1.771, 26.501)], and concurrent metabolic syndrome [OR 3.975, 95% CI (1.144, 13.815)] were risk factors for DR in diabetes without hypertension, and elevated HbA1c [OR 1.395, 95% CI (1.183, 1.645)], HbA1c ≥6.5% [OR 1.745, 95% CI (1.027, 2.966)], use of antidiabetic drugs [OR 4.781, 95% CI (2.624, 8.711)], elevated fasting blood glucose [OR 1.146, 95% CI (1.034, 1.270)], elevated postprandial blood glucose [OR 1.083, 95% CI (1.020, 1.151)], and nonspecific ST-T segment changes on electrocardiography [OR 2.616, 95% CI (1.531, 4.469)] were risk factors for DR in diabetes with hypertension.

**Conclusion:**

Retinopathy was found in subjects with normal glucose tolerance and prediabetes. There were differences in risk factors for DR in diabetic patients with and without hypertension.

## Introduction

The prevalence of diabetes in China has increased significantly in the past 30 years. The prevalence of diabetes and impaired glucose tolerance (IGT) in the population aged 25-64 years was 2.51% and 3.20%, respectively, in 1994 (1). The prevalence of diabetes in the population over 18 years in China reached 11.2% in 2020 (2). Diabetic retinopathy (DR) is a common chronic complication of diabetes, and it is the leading cause of irreversible vision loss in the working population (3, 4). The risk factors for DR include the duration of diabetes, hyperglycemia, hypertension, dyslipidemia and pregestational diabetes mellitus (3, 5, 6). Urbanization, the lack of timely fundus examinations, smoking, puberty, and subclinical hypothyroidism are also associated with the development of DR (7).

Previous studies showed that 95% of insulin-dependent diabetes patients and 58% of noninsulin-dependent diabetes patients were diagnosed with DR after 15 years of diabetes (8). A meta-analysis showed that the prevalence of diabetic retinopathy in the Chinese population with diabetes was 18.45%, and DR is becoming a serious social burden in China (9). Therefore, early diagnosis and intervention for modifiable risk factors for DR are important to reduce DR-induced blindness. The present study performed a cross-sectional survey of permanent residents aged over 40 years old in a community in Songjiang district, Shanghai. All residents with different types of abnormal glucose metabolism were evaluated for metabolic characteristics and fundus screening to examine the prevalence and risk factors for DR.

## Materials and methods

### Subjects and ethical approval

A total of 6,029 local residents from a community in Songjiang district, Shanghai, aged over 40 years and without hypothyroidism, hyperthyroidism, chronic renal failure, or excessive drinking (an alcohol intake > 140 g/week for men or >70 g/week for women) were enrolled in this cross-sectional survey. The Committee on Human Research at Shanghai General Hospital, Shanghai Jiao Tong University School of Medicine approved the study protocol. Written informed consent was obtained from each participant.

### Questionnaire and physical examination

Trained research staff performed standardized interviews of all patients that included a detailed questionnaire, anthropometry index, medical history, family histories of chronic diseases, smoking and drinking status, and current medication use.

All subjects underwent a physical examination in a fasting state. Blood pressure was measured three times consecutively, and the average of the three measurements was recorded for all subjects. Height, weight, waist circumference (WC), and hip circumference (HC) were measured in standing subjects. WC was measured midway between the lower edge of the costal arch and the top of the iliac crest. HC was measured around the widest portion of the buttocks. Body mass index (BMI) and the waist-to-hip ratio (WHR) were calculated after the data were collected.

### Laboratory tests and medical examination

Venous blood samples were collected from all participants the morning after an overnight fast of at least 10 hours. Subjects without a diagnosis of diabetes underwent the 75-g oral glucose tolerance test (OGTT), and subjects previously diagnosed with diabetes underwent the steamed bread meal test. Plasma glucose concentrations, uric acid (UA), total cholesterol (TCH), low-density lipoprotein cholesterol (LDL-C), high-density lipoprotein cholesterol (HDL-C), and triglycerides (TG) were measured enzymatically using an automatic biochemistry analyzer.

All subjects underwent electrocardiography. Fundus photographs were taken of both eyes of each participant using a nonmydriatic fundus digital retinal camera. Two ophthalmologists assessed the fundus using the fundus photographs.

### Diagnostic criteria

Diabetes and prediabetes were defined based on the 1999 World Health Organization (WHO) diagnostic criteria (10). The subjects were divided into three groups according to the results of the OGTT: normal glucose tolerance (Normal) group (Fasting plasma glucose (FPG)<6.1 mmol/L and 2hPG<7.8 mmol/L); prediabetes group (Impaired fasting glucose (IFG): FPG 6.1-6.9 mmol/L and 2hPG<7.8 mmol/L and impaired glucose tolerance (IGT): FPG<7.0 mmol/L and 7.8 ≤ 2hPG<11.1 mmol/L); and diabetes group (FPG≥ 7.0 mmol/L or/and 2hPG≥11.1 mmol/L).

Hypertension was defined as office systolic blood pressure (SBP) values ≥140 mmHg and/or diastolic blood pressure (DBP) values ≥90 mmHg and a documented diagnosis of hypertension and/or current use of antihypertensive agents (11).

Metabolic syndrome was defined according to the IDF criteria (12): elevated WC [defined as WC≥90 cm for males or ≥ 80 cm for females for an Asian population (13)] plus any two or more of the following criteria: 1) TG≥1.7 mmol/L or specific treatment for this lipid abnormality; 2) reduced HDL-C<1.03 mmol/L in males and <1.29 mmol/L in females or specific treatment for this lipid abnormality; 3) blood pressure ≥130/85 mmHg or treatment of previously diagnosed hypertension; and 4) fasting plasma glucose≥5.6 mmol/l or previously diagnosed type 2 diabetes.

### Statistical analysis

All statistical analyses were performed using SAS version 9.4 (SAS Institute Inc., Cary, NC, USA). Continuous data are presented as the means ± standard deviation (SD) and were compared using independent-samples t-tests or one-way ANOVA. Categorical variables were compared using the chi-squared test. Unconditional logistic regression models were used to calculate odds ratios (ORs) and 95% confidence intervals (CIs) for the variables. All P values were two-tailed, and a P value < 0.05 was considered statistically significant.

## Results

### Comparison of the demographic and clinical characteristics by glucometabolic condition

A total of 6,029 subjects were divided into three groups according to their glucometabolic conditions. A total of 1,349 subjects (22.38%) were included in the diabetes group, 1,482 subjects (24.58%) were included in the prediabetes group, and 3,198 subjects (53.04%) were included in the normal glucose tolerance group. The demographic and clinical characteristics of the three groups are shown in **Table 1**. The diabetes group were the oldest and had the highest rates of overweight (BMI≥24), central obesity (waist-to-hip ratio, female ≥ 0.85 male ≥ 0.9), elevated waist circumference (≥ 80 cm in female and ≥ 90 cm in male) and prevalence of hypertension and metabolic syndrome (all Ps<0.001). The levels of fasting blood glucose, postprandial blood glucose, HbA1c, TCH and TG in the diabetes group were also the highest of the three groups (all Ps<0.001). The prediabetes group had the highest serum UA level and rate of abnormal UA (UA ≥420 µmol/L) (P<0.001). The highest eGFR was in the normal group (P<0.001). There was no significant difference in the incidence of nonspecific ST-T segment changes on electrocardiography between the three groups (**Table 1**).

**Table 1:** Demographic and physical parameters of the participants in different glucose metabolism groups.

Parameters	Normal (n=3198)	Prediabetes (n=1482)	Diabetes (n=1349)	*P*
Age (years)	58.9 ± 8.8	61.7 ± 9.3	62.4 ± 8.7	**<0.001**
				**<0.001**
<55	1211 (37.9%)	401 (27.1%)	285 (21.1%)	
55-65	1246 (39%)	559 (37.7%)	579 (42.9%)	
≥65	741 (23.2%)	522 (35.2%)	485 (36.0%)	
Sex (%)				**0.003**
female	1237 (55.5%)	536 (52.5%)	524 (49.2%)	
male	990 (44.5%)	484 (47.5%)	541 (50.8%)	
SBP (mmHg)	131.9 ± 93.3	134.9 ± 15.6	139.3 ± 34.2	**0.006**
DBP (mmHg)	81.9 ± 18.9	83.2 ± 8.8	83.9 ± 9.0	**<0.001**
Hypertension				**<0.001**
no	1639 (52.5%)	563 (38.6%)	322 (24.4%)	
yes	1484 (47.5%)	895 (61.4%)	997 (75.6%)	
BMI (kg/m2)				**<0.001**
<24	1501 (48.0%)	524 (36.2%)	374 (28.6%)	
≥24	1627 (52.0%)	924 (63.8%)	934 (71.4%)	
Waist-hip ratio				**<0.001**
female<0.85 male<0.9	995 (44.7%)	385 (37.7%)	366 (34.4%)	
female≥0.85 male≥0.9	1232 (55.3%)	635 (62.3%)	699 (65.6%)	
Waist circumference (cm)				**<0.001**
female <80 male <90	1193 (54.8%)	465 (46.8%)	429 (41.7%)	
female ≥80 male ≥90	985 (45.2%)	528 (53.2%)	600 (58.3%)	
FBG (mmol/L)	5.3 ± 0.4	5.9 ± 0.6	7.8 ± 2.2	**<0.001**
PBG (mmol/L)	5.8 ± 1.2	8.1 ± 1.6	13.0 ± 4.6	**<0.001**
HbA1c (%)	5.5 ± 0.4	5.6 ± 0.4	6.6 ± 1.3	**<0.001**
eGFR [ml/ (min*1.73m2)]	92.2 ± 12.6	90.7 ± 12.6	91.5 ± 13.4	**0.01**
TCH (mmol/L)	5.1 ± 1.0	5.2 ± 1.0	5.3 ± 1.1	**<0.001**
TG (mmol/L)	1.32 ± 0.85	1.58 ± 1.24	1.87 ± 1.83	**<0.001**
LDL (mmol/L)	3.05 ± 0.79	3.11 ± 0.83	3.12 ± 0.88	**0.009**
HDL (mmol/L)	1.58 ± 0.43	1.55 ± 0.44	1.49 ± 0.43	**<0.001**
UA (μmol/L)	306.5 ± 80.6	323.1 ± 83	321.8 ± 82.9	**<0.001**
				**0.001**
<420	2902 (90.7%)	1295 (87.4%)	1186 (88.0%)	
≥420	296 (9.3%)	187 (12.6%)	162 (12.0%)	
Metabolic syndrome (%)				**<0.001**
no	1771 (83.2%)	652 (66.5%)	529 (51.7%)	
yes	358 (16.8%)	329 (33.5%)	494 (48.3%)	
Nonspecific ST-T segment change (%)				
no	2609 (81.6%)	1178 (79.5%)	1079 (80.0%)	0.179
yes	589 (18.4%)	304 (20.5%)	270 (20.0%)	

BMI, body mass index; SBP, systolic blood pressure; DBP, diastolic blood pressure; FBG, fasting blood sugar; HbA1c, glycosylated hemoglobin; HDL, high-density lipoprotein cholesterol; LDL, low-density lipoprotein cholesterol; PBG, postprandial blood glucose; TC, total cholesterol; TG, triglyceride. P < 0.05 were shown in bold values.

### Incidence of retinopathy by glucometabolic condition

**Figure 1:**
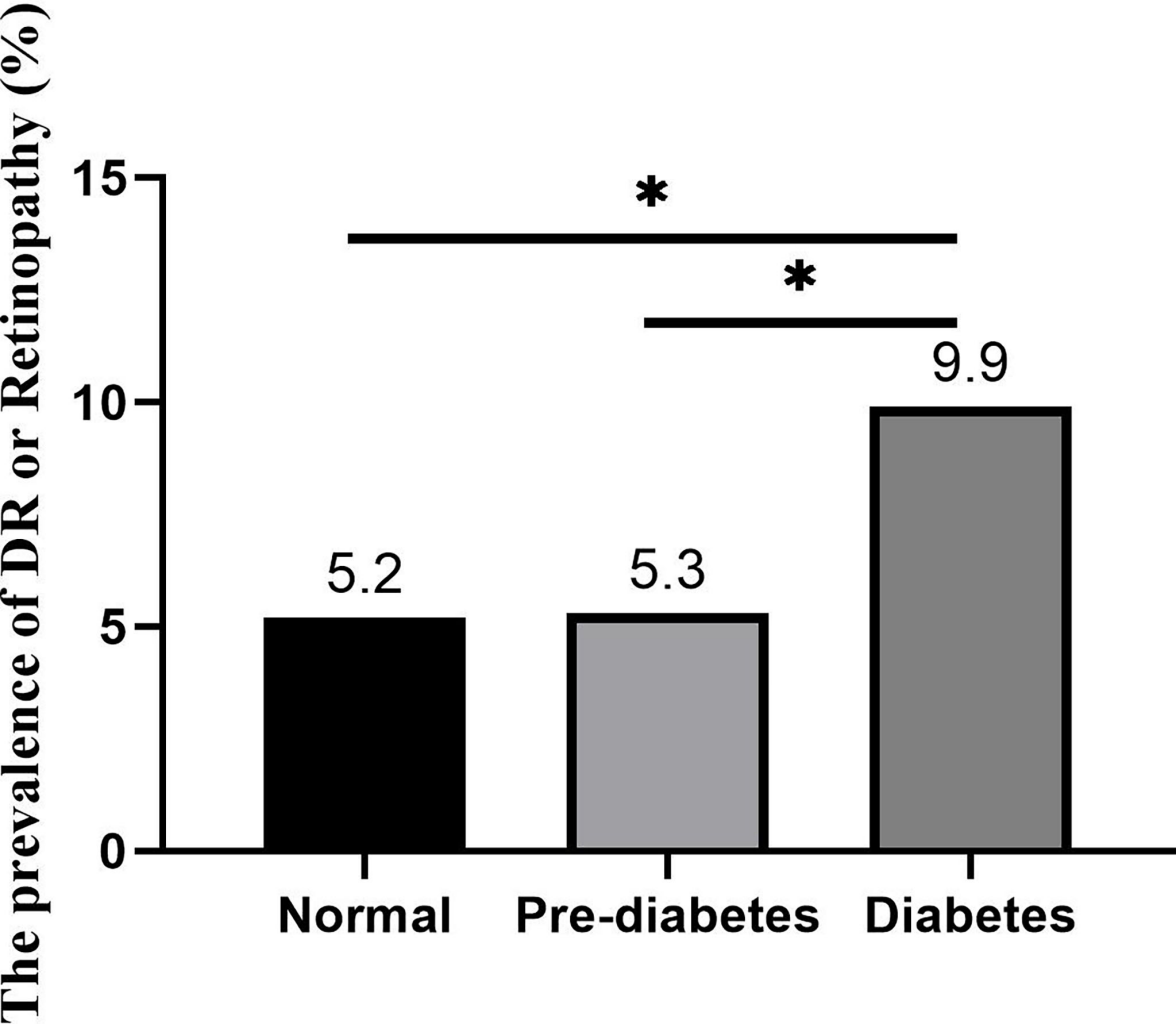
The prevalence of DR and retinopathy in different glucose metabolism groups *P<0.05.

As shown in **Figure 1**, the incidence of DR in the diabetes group was 9.9%. The incidence of DR in the prediabetes group and the normal group was 5.3% and 5.2%, respectively. The incidence rate of DR between the three groups was statistically significant (P<0.001).

### Risk factors for diabetic retinopathy in diabetes

The diabetes patients were divided into two groups, the nondiabetic retinopathy group (NDR group, 1,216 subjects) and the diabetic retinopathy group (DR group, 133 subjects). The results showed that duration of disease [OR 1.094, 95% CI (1.056, 1.133)], disease duration≥5 years [OR 3.104, 95% CI (2.099, 4.591)], WHR (≥0.85 in women, ≥0.9 in men) [OR 1.615, 95% CI (1.007, 2.590)], elevated HbA1c [OR 1.531, 95% CI (1.375, 1.703)], HbA1c≥6.5% [OR 2.792, 95% CI (1.919, 4.064)], the use of antidiabetic drugs [OR 3.485, 95% CI (2.395, 5.070)], elevated fasting blood glucose [OR 1.276, 95% CI (1.195, 1.363)], elevated postprandial blood glucose [OR 1.124, 95% CI (1.082, 1.168)] and nonspecific ST-T wave changes on electrocardiography [OR 1.626, 95% CI (1.084, 2.439)] were risk factors for DR, and age [OR 0.977, 95% CI (0.957, 0.999)] was a protective factor against DR.

After adjusting for age, sex, duration, antidiabetic drugs, blood pressure, WC and WHR, central obesity (WHR, women ≥0.85 and men ≥0.9) [OR 1.683, 95% CI (1.016, 2.790)], SBP≥140 mmHg [OR 1.875, 95% CI (1.158, 3.034)], elevated HbA1c [OR 1.410, 95% CI (1.220, 1.629)], HbA1c≥6.5% [OR 2.149, 95% CI (1.320, 3.498)], the use of antidiabetic drugs [OR 3.798, 95% CI (2.209, 6.529)], elevated fasting blood glucose [OR 1.176, 95% CI (1.072, 1.289)], elevated postprandial blood glucose [OR 1.090, 95% CI (1.033, 1.150)], and nonspecific ST-T segment changes on electrocardiography [OR 2.555, 95% CI (1.556, 4.196)] were risk factors for DR, and age [OR 0.965, 95%CI (0.938, 0.993)] remained a protective factor against DR (**Table 2**).

**Table 2:** Risk factors for DR in people with diabetes.

Parameters	NDR (n=1216)	DR (n=133)	*P*	OR (95% CI)	OR (95% CI)*
Age (years)					
	62.6 ± 8.8	60.9 ± 7.9	**0.037**	**0.977 (0.957, 0.999)**	**0.965 (0.938, 0.993)**
			0.301		
<55	250 (20.6)	35 (26.3)		REF.	REF.
55-65	525 (43.2)	54 (40.6)		0.735 (0.468, 1.153)	0.679 (0.385, 1.195)
≥65	441 (36.3)	44 (33.1)		0.712 (0.445, 1.140)	0.573 (0.311, 1.053)
DM duration (years)	3.3 ± 3.5	5.1 ± 4.9	**<0.001**	**1.094 (1.056, 1.133)**	1.043 (0.991, 1.089)
			**<0.001**		
<5	1039 (85.4)	87 (65.4)		REF.	REF.
≥5	177 (14.6)	46 (34.6)		**3.104 (2.099, 4.591)**	1.597 (0.845, 3.019)
Sex (%)			0.951		
female	475 (49.2)	49 (49.5)		REF.	REF.
male	491 (50.8)	50 (50.5)		0.987 (0.653, 1.493)	1.000 (0.646, 1.549)
SBP (mmHg)	139.3 ± 35.7	139.5 ± 16.1	0.931	1.000 (0.995, 1.005)	1.002 (0.997, 1.007)
			0.164		
<140	603 (52.2)	60 (45.8)		REF.	REF.
≥140	552 (47.8)	71 (54.2)		1.293 (0.900, 1.858)	**1.875 (1.158, 3.034)**
DBP (mmHg)	83.9 ± 9.2	84 ± 7.6	0.874	1.001 (0.982, 1.022)	1.003 (0.977, 1.031)
			0.884		
<90	804 (69.6)	92 (70.2)		REF.	REF.
≥90	351 (30.4)	39 (29.8)		0.971 (0.654, 1.441)	1.009 (0.616, 1.652)
Hypertension			0.335		
no	185 (24.0)	37 (27.8)		REF.	REF.
yes	901 (76.0)	96 (72.2)		0.821 (0.549, 1.227)	0.914 (0.557, 1.501)
BMI (kg/m2)			0.364		
<24	341 (29.0)	33 (25.2)		REF.	REF.
≥24	836 (71.0)	98 (74.8)		1.211 (0.801, 1.833)	1.1236 (0.719, 2.125)
Waist-hip ratio			**0.045**		
female<0.85 male<0.9	341 (35.3)	25 (25.3)		REF.	REF.
female≥0.85 male≥0.9	625 (64.7)	74 (74.8)		**1.615 (1.007, 2.590)**	**1.683 (1.016, 2.790)**
Waist circumference (cm)			0.068		
female <80 male <90	397 (42.6)	32 (33.0)		REF.	REF.
female ≥80 male ≥90	535 (57.4)	65 (67.0)		1.507 (0.968, 2.346)	1.573 (0.878, 2.819)
HbA1c (%)	6.5 ± 1.2	7.6 ± 1.9	**<0.001**	**1.531 (1.375, 1.703)**	**1.410 (1.220, 1.629)**
			**<0.001**		
<6.5	722 (59.6)	46 (34.6)		REF.	REF.
≥6.5	489 (40.4.7)	87 (65.4)		**2.792 (1.919, 4.064)**	**2.149 (1.320, 3.498)**
Hypoglycemic drugs			**<0.001**		
no	1006 (82.7)	77 (57.9)	1	REF.	REF.
yes	210 (17.3)	56 (42.1)		**3.485 (2.395, 5.070)**	**3.798 (2.209, 6.529)**
FBG (mmol/L)	7.6 ± 2	9.2 ± 3	**<0.001**	**1.276 (1.195, 1.363)**	**1.176 (1.072, 1.289)**
PBG (mmol/L)	12.8 ± 4.5	15.5 ± 4.9	**<0.001**	**1.124 (1.082, 1.168)**	**1.090 (1.033, 1.150)**
eGFR [ml/(min*1.73m2)]	91.5 ± 13.3	91.6 ± 15	0.928	1.001 (0.985, 1.016)	0.989 (0.970, 1.008)
TCH (mmol/L)	5.3 ± 1.1	5.1 ± 1.1	**0.041**	0.835 (0.703, 0.993)	0.830 (0.673, 1.024)
TG (mmol/L)	1.9 ± 1.8	1.9 ± 1.8	0.856	1.009 (0.918, 1.109)	1.044 (0.931, 1.170)
HDL (mmol/L)	1.5 ± 0.4	1.4 ± 0.4	0.139	0.717 (0.461, 1.114)	0.960 (0.555, 1.661)
LDL (mmol/L)			0.254		
≤1.8	60 (5.2)	10 (7.5)		REF.	REF.
>1.8	1103 (94.8)	123 (92.5)		0.669 (0.334, 1.341)	0.813 (0.324, 2.039)
UA (μmol/L)	322 ± 82	320.3 ± 90.9	0.824	1.000 (0.998, 1.002)	1.001 (0.999, 1.004)
			0.783		
<420	1068 (87.9)	118 (88.7)		REF.	REF.
≥420	147 (12.1)	15 (11.3)		0.924 (0.525, 1.624)	1.317 (0.654, 2.654)
Metabolic syndrome			0.081		
no	487 (52.6)	42 (43.3)		REF.	REF.
yes	439 (47.4)	55 (56.7)		1.453 (0.953, 2.215)	1.439 (0.839, 2.469)
Nonspecific ST-T segment change (%)			**0.018**		
no	983 (80.8)	96 (72.2)		REF.	REF.
yes	233 (19.2)	37 (27.8)		**1.626 (1.084, 2.439)**	**2.555 (1.556, 4.196)**

BMI, body mass index; SBP, systolic blood pressure; DBP, diastolic blood pressure; FBG, fasting blood sugar; HbA1c, glycosylated hemoglobin; HDL, high-density lipoprotein cholesterol; LDL, low-density lipoprotein cholesterol; PBG, postprandial blood glucose; TC, total cholesterol; TG, triglyceride;

*Adjusted for age, sex, diabetes duration, hypoglycemic drugs, hypertension, waist circumference and waist-hip ratio. P < 0.05 were shown in bold values.

### Risk factors for diabetic retinopathy in diabetes patients with and without hypertension

Hypertension is one of the most common comorbidities in diabetes. Diabetes was divided into two groups, with hypertension (n=997, 75.6%) and without hypertension (n=322, 24.4%).After adjusting for age, sex, duration, antidiabetic drugs, blood pressure, WC and WHR, the results showed that duration of diabetes [OR 1.206, 95% CI (1.028, 1.415)], central obesity (WHR ≥0.85 in women, ≥0.9 in men) [OR 3.796, 95% CI (1.144, 12.603)], elevated WC [OR 6.874, 95% CI (1.403, 33.665)], elevated HbA1c [OR 1.435, 95% CI (1.046, 1.970)], HbA1c≥6.5% [OR 6.850, 95% CI (1.771, 26.501)], and concurrent metabolic syndrome [OR 3.975, 95% CI (1.144, 13.815)] were risk factors for DR in diabetes patients without hypertension. Elevated HbA1c [OR 1.395, 95% CI (1.183, 1.645)], HbA1c ≥6.5% [OR 1.745, 95% CI (1.027, 2.966)], the use of antidiabetic drugs [OR 4.781, 95% CI (2.624, 8.711)], elevated fasting blood glucose [OR 1.146, 95% CI (1.034, 1.270)], elevated postprandial blood glucose [OR 1.083, 95% CI (1.020, 1.151)], and nonspecific ST-T segment changes on electrocardiography [OR 2.616, 95% CI (1.531, 4.469)] were risk factors for DR in diabetes with hypertension (**Table 3**).

**Table 3:** Risk factors for DR in diabetic patients with and without hypertension.

Parameters	Diabetic patients with hypertension (n =997)		*P*	OR (95% CI)*	Diabetic patients without hypertension (n =322)		*P*	OR (95% CI)*
	NDR (n=901)	DR (n=96)			NDR (n=285)	DR (n=37)		
Age (years)	63.5 ± 8.8	62.3 ± 7.6	0.212	0.967 (0.938, 0.996)	60.0 ± 8.1	57.3 ± 7.6	0.061	0.957 (0.879, 1.042)
Age (years)			0.791				**0.044**	
<55	156 (17.3)	17 (17.7)		REF.	84 (29.5)	18 (48.7)		REF.
55-65	385 (42.7)	44 (45.8)		0.764 (0.393, 1.489)	128 (44.9)	10 (27.0)		0.374 (0.108, 1.288)
≥65	360 (40.0)	35 (36.5)		0.598 (0.297, 1.204)	73 (25.6)	9 (24.3)		0.722 (0.178, 2.926)
Diabetic duration (years)	3.5 ± 3.8	5.5 ± 5.1	**<0.001**	1.021 (0.964, 1.080)	2.8 ± 2.3	4.3 ± 4.3	0.052	**1.206 (1.028, 1.415)**
Diabetic duration (years)			**<0.001**				**0.039**	
<5	762 (84.6)	28 (61.5)		REF.	252 (88.4)	28 (75.7)		REF.
≥5	139 (15.4)	37 (38.5)		1.342 (0.664, 2.714)	33 (11.6)	9 (24.3)		2.447 (0.575, 10.415)
Sex (%)			0.562				0.380	
female	379 (49.1)	42 (52.5)		REF.	83 (47.4)	7 (36.8)		REF.
male	393 (50.9)	38 (47.5)		0.844 (0.517, 1.375)	92 (52.6)	12 (63.2)		1.167 (0.396, 3.434)
Waist-hip ratio			0.359				**0.020**	
female<0.85 male<0.9	241 (31.2)	21 (26.3)		REF.	86 (49.1)	4 (21.1)		**REF.**
female≥0.85 male≥0.9	531 (68.8)	59 (73.8)		1.183 (0.683, 2.048)	89 (60.9)	15 (79.0)		**3.796 (1.144, 12.603)**
Waist circumference (cm)			0.49				**0.006**	
female <80 male <90	286 (38.1)	27 (34.2)		REF.	104 (61.2)	5 (27.8)		**REF.**
female ≥80 male ≥90	464 (61.9)	52 (65.8)		1.093 (0.580, 2.058)	66 (38.8)	13 (72.2)		**6.874 (1.403, 33.665)**
HbA1c (%)	6.5 ± 1.1	7.6 ± 1.9	**<0.001**	**1.395 (1.183, 1.645)**	6.7 ± 1.4	**7.6 ± 2**	**0.005**	**1.435 (1.046, 1.970)**
HbA1c (%)			**<0.001**				**<0.001**	
<6.5	538 (60.0)	35 (36.5)		**REF.**	170 (59.9)	11 (29.7)		**REF.**
≥6.5	359 (40.0)	61 (63.5.0)		**1.745 (1.027, 2.966)**	114 (40.1)	26 (70.3)		**6.850 (1.771, 26.501)**
Hypoglycemic drugs			**<0.001**				0.075	
no	735 (81.6)	49 (51.0)		**REF.**	247 (86.7)	28 (75.7)		REF.
yes	166 (18.4)	47 (49.0)		**4.781 (2.624, 8.711)**	38 (13.3)	9 (24.3)		1.137 (0.281, 4.608)
FBG (mmol/L)	7.6 ± 2	9.2 ± 2.9	**<0.001**	**1.146 (1.034, 1.270)**	7.7 ± 2.1	9.2 ± 3.1	**0.005**	1.215 (0.976, 1.513)
PBG (mmol/L)	12.7 ± 4.4	15.5 ± 4.8	**<0.001**	**1.083 (1.020, 1.151)**	12.8 ± 4.9	15.6 ± 5.2	**0.003**	1.094 (0.971, 1.234)
TCH (mmol/L)	5.3 ± 1.1	5 ± 1.2	**0.012**	0.801 (0.634, 1.010)	5.2 ± 1.0	5.3 ± 1.0	0.714	1.145 (0.729, 1.799)
TG (mmol/L)	1.9 ± 1.7	1.9 ± 2	0.97	1.066 (0.940, 1.208)	1.8 ± 1.9	1.9 ± 1.5	0.737	0.894 (0.585, 1.366)
HDL (mmol/L)	1.5 ± 0.4	1.5 ± 0.4	0.468	1.039 (0.574, 1.880)	1.5 ± 0.4	1.4 ± 0.4	0.126	1.088 (0.303, 3.906)
LDL (mmol/L)			0.254				0.658	
≤1.8	47 (5.5)	8 (8.3)		REF.	11 (4.0)	2 (5.4)		NA
>1.8	812 (94.5)	88 (91.7)		0.676 (0.264, 1.735)	263 (96.0)	35 (94.6)		NA
UA (μmol/L)			0.978				0.758	
<420	296 (38.6)	31 (38.8)		REF.	260 (91.2)	33 (89.2)		NA
≥420	471 (61.4)	49 (61.3)		0.628 (0.348, 1.132)	25 (8.8)	4 (10.8)		NA
Metabolic syndrome (%)			0.571				**0.003**	
no	347 (46.4)	34 (43.0)		REF.	132 (78.6)	8 (44.4)		REF.
yes	401 (53.6)	45 (57.0)		1.056 (0.584, 1.912)	36 (21.4)	10 (55.7)		**3.975 (1.144, 13.815)**
Nonspecific ST-T segment change (%)			**0.013**				0.631	
no	710 (78.8)	65 (67.7)		REF.	247 (85.7)	31 (83.8)		REF.
yes	191 (21.2)	31 (32.3)		**2.616 (1.531, 4.469)**	38 (13.3)	6 (16.2)		1.960 (0.461, 8.336)

BMI, body mass index; SBP, systolic blood pressure; DBP, diastolic blood pressure; FBG, fasting blood sugar; HbA1c, glycosylated hemoglobin; HDL, high-density lipoprotein cholesterol; LDL, low-density lipoprotein cholesterol; PBG, postprandial blood glucose; TC, total cholesterol; TG, triglyceride. NA means Not Available.

*Adjusted for age, sex, diabetes duration, hypoglycemic drugs, hypertension, waist circumference and waist-hip ratio. P < 0.05 were shown in bold values.

## Discussion

The prevalence of diabetes is 11.2% in Chinese adults, but it is approximately 20% in the population over 60 years old (2). The rapidly increasing prevalence of diabetes in China may be attributed to urbanization, aging, and overweight/obesity, and people living in economically developed areas and urban areas had a higher prevalence of diabetes (13–15). The present study performed systematic physical examinations on 6,029 permanent residents over the age of 40 years living in the community in Songjiang district, Shanghai. Similar to the results of previous studies, the prevalence of diabetes and prediabetes in the community population reached 22.38% and 24.58%, respectively. Comparisons of the demographic and clinical characteristics of diabetes, prediabetes and normal glucose tolerance subjects in this study revealed that the diabetes group was older, had a higher proportion of overweight and central obesity, and higher blood lipid levels. The proportion of patients with comorbid hypertension and metabolic syndrome was also higher. These results are consistent with previous studies (2, 15).

DR is the most common microvascular complication of diabetes (16). DR is the leading cause of irreversible vision loss in the working population (3, 4). In this study, we used nonmydriatic fundus digital retinal camera to screen the DR. Seven field mydriatic color fundus photography, which could record both of central and peripheral retinal lesions, has been considered the reference standard for diagnosing DR (17). But pharmacologic pupil dilation may cause discomfort to the subject and raise the risk of angle closure glaucoma, making it inappropriate for community DR screening. The sensitivity for detection of DR with a single field non-mydriatic scan image varies from 64 to 98% (18). Lin et al. reported highly significant agreement between the degree of diabetic retinopathy detected by a single nonmydriatic monochromatic digital photo graph of central retina and that seen in seven standard color mydriatic fields (19). Compared to the mydriatic fundus oculi with ophthalmoscopy, that retinal photography is more cost-effective.

The prevalence of DR is 25.08% worldwide (20). A meta-analysis showed that the prevalence of DR was 18.45% in China (9). Zhang P et al. found that the prevalence of DR in Shanghai was 16.97% (21). The present study found that the prevalence of DR in community patients with diabetes was 9.9%, which was lower than reported in previous studies. There are many associated factors affecting the prevalence of DR. Patients in economically developed areas have a higher incidence of diabetes but a lower incidence of DR (22, 23). The risk of DR increases with the duration of diabetes (24). Most subjects in our study were newly diagnosed with diabetes (62.5% of patients with diabetes) and lived on the outskirts of Shanghai, and these factors may partially explain the low prevalence of DR.

DR, especially proliferative DR, is generally considered a unique complication of diabetes. However, 7.3% to 18.2% of patients already have DR at the time of diabetes diagnosis, and a previous study reported that among patients with DM, DR might develop 4 to 7 years before the diagnosis of diabetes (25). Undiagnosed diabetes might have comparable DR risk among those with already known diabetes (26). Retinopathy lesions may represent early microvascular damage from a combination of insults by various risk factors and individual with different glucose metabolism status susceptibilities to such insults. The typical lesions of diabetic retinopathy (retinal microaneurysms, hemorrhages, and cotton wool spots) are commonly seen in persons without clinically diagnosed diabetes. Retinopathy was common among rural Chinese adults without diabetes. Its association with blood glucose and blood pressure suggests that early microvascular damage is occurring at “high normal” levels of blood glucose and blood pressure (27). In this suburban community-based cross-sectional study, a certain proportion of retinopathy was observed in subjects with prediabetes and normal glucose metabolism tolerance, and the prevalence rates were 5.3% and 5.2%, respectively. Therefore, fundus photograph examinations for populations without diabetes or high-risk populations with diabetes are necessary and helpful for the early diagnosis and intervention for DR or retinopathy. However, the diagnosis of DR is often late or absent in both suburban and rural populations. Telemedicine could allow for a specialist examination of the fundus oculi in short times and an early diagnosis of diabetic retinopathy, as well as the possibility to assess its progression (28, 29). Telemedicine may be of great help to the prevention and treatment of DR especially for the suburban and rural populations in the future.

Many factors are involved in the development of DR including the duration of diabetes, hyperglycemia, hypertension, dyslipidemia and pregestational diabetes mellitus (5, 6). In this study, central obesity (WHR, women ≥ 0.85, men ≥ 0.9), elevated HbA1c, HbA1c ≥ 6.5%, antidiabetic drug use, elevated fasting blood sugar, elevated postprandial blood sugar and nonspecific ST-T segment changes on electrocardiography were independent risk factors for DR after adjusting for relevant factors. Our results are consistent with previous studies. Yun Peng et al. found that duration, the use of antidiabetic drugs and diabetic peripheral neuropathy were independent risk factors for DR in a Shenzhen community in China (30). A high WHR is an indicator of abdominal obesity. Among Asian women with type 2 diabetes, researchers found that WHR was positively associated with mild/moderate [OR 3.49 (1.50, 8.10)] and severe [OR 2.68 (1.28, 5.62)] DR (31). One study in China showed that subjects in the highest tertile of WHRs at baseline had a significantly higher risk of DR during a six-year follow-up [OR 1.44, (1.17-1.78)] (32). Chronic hyperglycemia-mediated capillary damage is the pathophysiology of DR, and poorly controlled blood sugar and HbA1c have always been risk factors for DR (3). Many studies demonstrated that intensive treatment of glycemia slowed the progression of DR (33, 34). Previous studies also showed that poorly controlled hypertension was significantly associated with DR (35). We found that SBP ≥140 mmHg was a risk factor for DR, which suggests that poor control of SBP is more related to DR. The relationship between nonspecific ST-T wave changes which is the indicators for cardiovascular events and diabetic retinopathy has not been reported. Whether ST-T wave changes on electrocardiography can be used as a predictor of DR remains to be further confirmed by large prospective studies. We found that age was a protective factor against DR. Previous studies suggest that age is a risk factor for DR (36). The population aged 60-69 years had the highest prevalence of DR in China, which increased dramatically with the duration of diabetes (9). Therefore, the differences between our results and a previous study may be due to the age and diabetes duration of the subjects. In addition, it is well- known that patients with both a high albumin excretion rate (AER) and low glomerular filtration rate (GFR) at baseline will have the higher risk of diabetic retinopathy in type 2 diabetic patients (37). In this study, we didn’t measure the level of AER and the level of eGFR was not related to the DR.

Hypertension is one of the most common complications of diabetes. According to the 3B Study in China, the prevalence of hypertension in diabetes has reached 59.9% (38). The proportion of diabetes patients with hypertension reached 75.6% in our study. Therefore, we further examined the risk factors for DR in diabetes patients with and without hypertension. We found that elevated HbA1c and HbA1c≥6.5% were common risk factors for DR in diabetics with and without hypertension, which further suggests the importance of glycemic control in preventing DR. We also found that duration, elevated WHR, elevated WC and metabolic syndrome were risk factors for DR in diabetics without hypertension. As mentioned above, abdominal obesity plays an important role in the development of DR, and type 2 diabetes patients with a low BMI but a higher WHR had a significantly increased risk of developing DR (32). Metabolic syndrome is a risk factor for DR in females in China (39). Our findings suggest that reducing abdominal obesity and metabolic syndrome in diabetes patients without hypertension will help slow the process of DR. Interestingly, our study suggests that nonspecific ST-T segment (diabetic macrovascular complication) may also be a predictor of DR (diabetic microvascular complication) in diabetic patients with hypertension. Although hypertension is one of the established risk factors of diabetic retinopathy, the relationship between hypertension and DR remains unclear in different studies (40). Previous studies have suggested that a comorbidity of hypertension is a high-risk factor leading to cardiovascular events in diabetes (41). This study aimed to investigate the associations of DR risk factors with or without hypertension, the results showed that more attention should be paid to centripetal obesity as well as general obesity for DR in diabetes without hypertension, while, in addition to elevated HbA1c, nonspecific ST-T segment changes on electrocardiography were risk factors for DR in diabetes with hypertension. Further research is also needed to focus on the improvement of hypertension management in patients with DR.

There are several potential limitations in our research. The present study was a cross-sectional observation, and no causal inferences may be drawn. Although we found several risk factors for DR, including central obesity, elevated SBP, poor glycemic control, nonspecific ST-T segment changes, and the duration of diabetes, it was difficult to identify any causal relationships. The findings of a single-center clinical study cannot be used for all DR populations in our country due to differences in economic and cultural development, urbanization, and geographic distribution. Based on the current cross-sectional research findings, multicenter prospective studies on a larger scale are required to clarify the causal associations of the levels of blood glucose, hypertension, nonspecific ST-T segment changes, and metabolic syndrome with DR in community residents.

## Conclusion

The prevalence of DR in diabetes patients in a community in Songjiang district, Shanghai, was 9.9%. Retinopathy was also found in prediabetes and normal glucose tolerance subjects. Therefore, fundus photograph examination for populations without diabetes or high-risk populations of diabetes may help with early diagnosis and intervention for DR. We also demonstrated that an elevated WHR, SBP≥140 mmHg, the use of antidiabetic drugs, poor blood glucose control and nonspecific ST-T segment changes on electrocardiography were risk factors for DR. Poor blood sugar control and nonspecific ST-T segment changes on electrocardiography are risk factors for DR in diabetics with hypertension. Poor blood sugar control, abdominal obesity and concurrent metabolic syndrome are risk factors for DR in diabetics without hypertension. Because the DR-induced vision loss is irreversible, intensive control of glycemia and blood pressure, reductions in abdominal obesity and other comprehensive management measures for metabolic syndrome may be important methods to delay the progression of DR and protect patients’ vision.

## Data availability statement

The raw data supporting the conclusions of this article will be made available by the authors, without undue reservation.

## Ethics statement

The studies involving human participants were reviewed and approved by Committee on Human Research at Shanghai General Hospital, Shanghai Jiao Tong University School of Medicine. The patients/participants provided their written informed consent to participate in this study.

## Author contributions

Conceptualization: ZZ, YP and XD. Methodology, data curation, and formal analysis: HW, JJ, CL, CH, and YW. Original draft: YM and SZ. Writing, review and editing: all authors. YM, HW, and JJ contributed equally to this work. All authors contributed to the article and approved the submitted version.

## Funding

The study was supported by the National Natural Science Foundation of China (81870596, 81870594); Clinical Research Plan of SHDC (No. SHDC2020CR1016B); Shanghai Jiao Tong University Research Funding on Medical, Engineering Interdisciplinary Project (YG2019GD05); Multicenter Clinical Research Project of Shanghai Jiao Tong University School of Medicine (DLY201824); Third Round Cooperation Project of Songjiang District Municipal Health Commission (0702N18003); and Shanghai General Hospital Clinical Research Innovation Team Project (CTCCR-2018A02).

## Acknowledgments

The authors acknowledge the contributions of all the participants.

## Conflict of interest

The authors declare that the research was conducted in the absence of any commercial or financial relationships that could be construed as a potential conflict of interest.

## Publisher’s note

All claims expressed in this article are solely those of the authors and do not necessarily represent those of their affiliated organizations, or those of the publisher, the editors and the reviewers. Any product that may be evaluated in this article, or claim that may be made by its manufacturer, is not guaranteed or endorsed by the publisher.
